# Opportunities to strengthen resilience of health care workers regarding patient safety

**DOI:** 10.1186/s12913-023-10054-0

**Published:** 2023-10-19

**Authors:** Veronika Pacutova, Andrea Madarasova Geckova, Andrea F. de Winter, Sijmen A. Reijneveld

**Affiliations:** 1 Department of Health Psychology and Research Methodology, Faculty of Medicine, P. J. Safarik University, Trieda SNP 1, Kosice, 040 11 Slovakia; 2 Department of Community & Occupational Medicine, University Medical Center Groningen, University of Groningen, Groningen, 9713 Netherlands; 3 Institute of Applied Psychology, Faculty of Social and Economic Sciences, Comenius University Bratislava, Bratislava, 821 05 Slovakia

**Keywords:** Health care workers, Patient safety, Hospital management, COVID-19, Second victim

## Abstract

**Background:**

The COVID-19 pandemic endangered the quality of health care and the safety of patients and health care workers (HCWs). This provided challenges for HCWs’ resilience and for hospital management and probably increased risks for patient safety incidents (PSI). HCWs may also have experienced psychological consequences as second victims of PSI, but evidence on this is lacking. Therefore, we mapped HCWs’ experiences with PSI during the second wave of COVID-19, the associations of these experiences with the hospital management of patient safety culture and HCWs’ interests in receiving further training.

**Methods:**

We obtained data from 193 HCWs working at the COVID-related departments of one large hospital in eastern Slovakia via a questionnaire developed in direct collaboration with them. We measured PSI experiences as various HCWs’ experiences with near miss and adverse events and the hospital management of patient safety culture using indicators such as risk of recurrence, open disclosure and second victim experiences. For analysis, we used logistic regression models adjusted for age and gender of the HCWs.

**Results:**

One-third of the hospital HCWs had experienced PSI; these were more likely to expect adverse events to recur (odds ratio, OR = 2.7–3.5). Regarding the hospital management of patient safety culture, the HCWs’ experiencing openly disclosed PSI was associated with one negative outcome, i.e. conflicts among colleagues (OR = 2.8), and one positive outcome, i.e. patients’ acceptance of their explanation and apologies (OR = 2.3). We found no associations for any other essential domains after disclosure. PSI experiences were strongly associated with psychological indicators of second victimhood, such as sadness, irritability, anxiety and depression (OR = 2.2–4.3), while providing support was not. The majority of the HCWs would like to participate in the suggested trainings (83.4%).

**Conclusion:**

HCWs with PSI experiences reported poor hospital management of the patient safety culture, which might reflect they missed the opportunities to strengthen their resilience, especially during the COVID-19 pandemic.

**Supplementary Information:**

The online version contains supplementary material available at 10.1186/s12913-023-10054-0.

## Introduction

The COVID-19 pandemic (hereinafter as the “the pandemic”) struck countries unexpectedly, and they were not prepared to fight against it and protect themselves, especially those without previous experience with similar epidemics, such as SARS and MERS. Health care workers (HCWs) faced many new occupational challenges, such as workload, exhaustion, staff inexperience, understaffing, poor leadership, impacted patient safety culture and stress and even classified it as “pandemic nursing care”, which is defined as “the rushed, physically overwhelming and emotionally draining care provided to an onslaught of critically ill patients.” [1–4]. All these factors endangered the quality and safety of health care by increasing the risk of patient safety incidents (PSI). Here, we consider PSI to be any event, which could have caused harm to a patient, such as adverse events (AEs), or potentially might have resulted in harm but did not, such as so-called “near misses” [5]. Factors making PSI more likely during the pandemic are related to the lack of experience of critical care staff, lack of experience with ventilator management, a high nurse-to-patient ratio, the use of personal protective equipment and the severity of patient’s condition. HCWs even felt blamed, targeted, discouraged, and deserted by hospital managers and leaders, because of their own inability to do something about the frequency of PSI [6, 7].

Hospital leaders are responsible for setting up the patient safety culture by creating a safe, open non-punitive supportive workspace for HCWs, making them feel free to openly communicate and report when something is wrong. There is thus a high demand on these leaders to build a support system for HCWs, in particular helping to strengthen their resilience and willingness to speak up for themselves [8]. However, hospital leaders reported feeling unqualified to help HCWs with their mental challenges and unprepared for taking responsibility for this [9]. Also, HCWs identified major barriers regarding hospital leadership in their process of seeking help, such as misunderstanding roles, a lack of leadership training, the perception of resilience as a personal and not an organisational concern and workplaces being socially unsafe [9].

The patient safety culture in Slovakia is poorly developed since there have long been significant differences between the private and public health care sectors caused by differences in hospital leadership [10–14]. The poor safety culture relates to issues regarding understaffing, the blame culture, a lack of open communication, the weakness of teamwork collaboration, burnout and unfinished nursing care (tasks left undone, missed care, and implicit rationing of nursing care) [14]. Hospital management is hardly promoting patient safety as a top priority and is interested in patient safety only after the occurrence of AEs [10–14]. Based on all the above-mentioned, Slovak HCWs reports that they are still afraid to report any PSI to hospital management [10]. However, despite the persisting fear of reporting, we noticed a four-fold increase in voluntary reporting of HCW errors one year after the pandemic, specifically in drug/treatment prescription and failure of attention, which was proved to be affected even by their own COVID-19 infection due to professional exposure [10, 15]. This increase in Slovak reports of medical errors might be due to a national law, where reporting without blame in the case of an emergency is a part of Slovak regulation [16]. We might consider their reporting as being freed from negative consequences (blaming, legal consequences, losing reputation/job). However, other countries have also reported an increasing rate of prescribing errors during the pandemic, with issues related to wrong doses or frequency of use [17, 18].

Usually, evidence regarding the effects of a poor safety culture relates to patients, but an impact on HCWs is also likely. Also, HCWs who are involved in unexpected AEs might be the victims of traumatic events, and we might use a term such as “second victim” (SV) [19]. The prevalence of the SV phenomena was reported by 59% of nurses in Germany and by 72.5% of hospital HCWs in Spain [20–22]. Therefore, we aimed to map the experiences of Slovak HCWs with PSI during the second pandemic wave, the association of these experiences with hospital management of patient safety culture (risk of recurrence, open disclosure/second victim experience) and describe their interests in receiving further training.

## Methods

### Sample and procedures

All practising HCWs from the COVID-related departments (e.g. infection/anaesthesiology and intensive care/pathology) of one hospital (covering the Kosice region), one rescue service (covering the Kosice region) and one dialysis service (covering the whole of Slovakia) were invited to participate in a cross-sectional study during the second wave of the pandemic through their employing organisation. We approached specifically those departments, which were primarily reprofiled in the first and second pandemic wave and explicitly those HCWs, who were assigned to COVID-19 department. In total, we received 233 responses, covering around 8% of the overall number of employees in the hospital. We were not able to compute a response rate, because we did not reach the HCWs personally; however, the invitation was disseminated through their employing organisation (bulletin boards, web, advertisements for employees). Afterwards, we excluded those who did not report their gender (n = 12), who did not specify their profession (n = 6) and whose profession was in a specific disease field (n = 22), which is not related to AEs reporting. Our final sample involved 193 respondents (80.8% females, mean age/SD = 45/±10.2).

Data were collected via a questionnaire, paper-based or online. This questionnaire was specifically established by HCWs’ representatives in direct interviews and based on a literature review. We designed applicable measurements to cover it, considering their opinions, and the final version was piloted in a small sample of HCWs to ensure accuracy and appropriateness. The questionnaire covered eleven diverse areas: sociodemographic information, exposure to COVID-19, risk perception/acceptance/stigma/vulnerability, information overload, non-adherence to pandemic measures, impact on health care provision, barriers and facilitators of health care provision, impact on quality of life (QoL), adverse events, help and support provided and personal coping resources. The questions on AEs were derived from a questionnaire used in a Spanish study by Jose Mira et al., with extra explanations of terms such as near miss and AEs provided to avoid HCWs’ possible misinterpretation [22]. This questionnaire is presented in Appendix A.

### Measures

Measures regarded patient safety incident experience (PSI experience) and hospital management of patient safety culture (risk of recurrence, open disclosure, SV experience and promotion of training). Question are shown, in full, in Appendix A.

#### Patient Safety Incident experience (PSI experience)

**PSI experience** was created by combining two questions: “Have you seen/heard about near misses/adverse events at your department/hospital in the last year?”, answers were yes vs. no. Our result variable covers those who saw/heard about “near misses” or “adverse events” at their department/hospital in the last year vs. those who did not see/hear.

#### Hospital management of patient safety culture

Hospital management of patient safety culture consists of Risk of Recurrence, Open disclosure/SV experience and Promotion of training. **Risk of recurrence** was measured by asking HCWs to indicate: How probable do you think there will be a next AE with serious consequences at your hospital/department? Answers were dichotomised as very likely/somewhat likely vs. unlikely.

**Open disclosure experience** regarded HCWs’ experiences in relation to institutional and patient responses to their speaking up about PSI and was measured by asking our HCWs what followed in the event of an error from their own experience or what they heard: (a) reporting/root-cause analysis, (b) informing and apologising to patient, (c) conflicts among colleagues, (d) losing reputation, (e) offering institutional support, (f) buddy support (by colleagues), (g) patient’s acceptance of explanation and apology, (h) patient’s rejection of apology, (i) patient complaint or (j) patient-HCW relation worsen. Answers were dichotomised as almost always/always vs. sometimes/never/does not concern them.

**SVs’ experiences** regarded HCWs’ psychological responses and was measured by asking HCWs how frequently they suffered from sadness, irritability, and anxiety in the last 6 months. Answers were dichotomised as every day/more than once in a week vs. every week/month/rarely/never. Regarding suicidal ideation, we asked them how many times in the last week they had thoughts they would be better off dead or of hurting themselves in some way. Answers were dichotomised as several days/more than a half the days/nearly every day vs. not at all. For monitoring depression, we used a standardised PHQ-9 questionnaire [23].

**Promotion of training** concerned HCWs’ interest in receiving specific training in: (a) how to communicate a PSI, (b) how to handle an uncooperative or aggressive patient, (c) how to inform a patient or family about AEs and (d) how HCWs could better cope with the aftermath of AEs. Answer options were yes vs. no.

#### Background characteristics

Background characteristics regarded age, gender, profession and job department of HCWs.

### Statistical analysis

First, we described the background of our sample using descriptive statistics. Second, we specified rates of PSI experiences and the hospital management of the patient safety culture (risk of recurrence, open disclosure/SV experience and promotion of training) among hospital HCWs. Third, we assessed the associations of PSI experiences with hospital management of patient safety culture using logistic regression, adjusted for age (continuous level variable, centred age and age squared) [24] and gender. This led to odds ratios (OR) with the 95% confidence intervals (CI) for each outcome. All analyses were performed using IBM SPSS Statistics 23 for Windows.

## Results

### Background characteristics

The majority of HCWs in our sample were females, 80.8% (n = 156), and more than a half were nurses, 58.0% (n = 112); 25.4% (n = 49) were doctors. For more details, see Table 1.

**Table 1:** Demographic characteristics of the HCWs (Slovakia 2021; n = 193 HCWs).

Variables	N (%)
**Age (mean/SD)**	45.0/±10.2
**Gender**	
Women	156 (80.8)
Men	37 (19.2)
**Profession**	
Nurses	112 (58.0)
Doctors	49 (25.4)
Rescuers	27 (14.0)
Other HCWs	5 (2.6)
**Department of HCWs**	
*Hospital – local*	
Infection department	46 (23.8)
Anaesthesiology and Intensive Care department	25 (13.0)
*Hospital – local and serving other hospitals*	
Dialysis department	92 (47.7)
Rescue department	30 (15.5)

### Rates of PSI experiences and hospital management of patient safety culture of HCWs

One-third of HCWs experienced at least one PSI, which means they saw or heard about a near miss or AE in their hospital during the last year. Further, more than half of HCWs expected the recurrence of an AE at their department or hospital, mostly at their hospital; and only few reported experiencing room for open disclosure. HCWs reported psychological consequences as SV, mostly depression, irritability, anxiety and even suicidal ideation (10 of all 14 were exactly those who had PSI experience). Finally, the majority of them expressed a high demand to be trained on how to better handle the patient and aftermath of AEs. For more details, see Table 2.

**Table 2:** Description of HCWs experiences of PSI and of hospital management of the patient safety culture

Variables	N (%)
**PSI experience**	
Saw/heard about near misses or AEs at their department/hospital in the last year	64(33.2)
**Hospital management of patient safety culture**	
• **Risk of recurrence**	
Expectation of next AE at department in the next 12 months^1^	79(41.8)
Expectation of next AE at hospital in the next 12 months^1^	107(56.0)
• **Open disclosure experience**	
***Institutional response***	
Reporting and root-cause analysis^2^	36(18.7)
Informing and apologising to patient^2^	35(18.1)
Conflicts among the colleagues^2^	25(13.0)
Losing reputation^2^	12( 6.2)
Offering institutional support^2^	12( 6.2)
Buddy support (by colleagues)^2^	56(29.0)
***Patient response***	
Accepting explanation and apology^2^	36(18.7)
Reject the apology and respond aggressively^2^	14( 7.3)
Patient filed a formal complaint^2^	7( 3.6)
Patient-HCW relation worsens^2^	20(10.4)
• **Second victim experience**	
Sadness^3^	31(16.1)
Irritability^3^	40(20.7)
Anxiety^3^	29(15.0)
Suicidal ideation^3^	14( 7.3)
Depression^4^	72(37.3)
* Mild (5–9)*	43(22.8)
* Moderate (10–14)*	11( 5.8)
* Moderate severe (15–19)*	12( 6.3)
* Severe (20–27)*	6( 3.2)
• **Promotion of training**	
How to communicate an PSI^5^	134(69.4)
How to handle an uncooperative or aggressive patient^5^	161(83.4)
How to inform patient or family about AEs^5^	135(69.9)
How could HCWs better cope with aftermath of AEs^5^	155(80.3)

^1^ very likely/somewhat likely; ^2^ always/almost always; ^3^ one or more days; ^4^ PHQ-9 score (overall); ^5^ yes

### Associations of PSI experience with the hospital management of patient safety culture

HCWs who experienced PSI were more likely to expect another AE at their department or at their hospital in the next 12 months (ORs varying between 2.7 and 3.5). HCWs reported that the required responses after PSI did not occur, such as reporting and root-cause analysis of AEs, informing of and apologising to the patient, and providing support to HCWs. Having a PSI experience made HCWs 2.8-times more likely to expect conflicts among colleagues; 2.3-times more likely to expect that patients accept explanations and apologies; and 2.2–4.5-times to expect to have the psychological consequences of an SV (such as sadness, irritability, anxiety and depression). Generally, age did not contribute, and gender only for risk of recurrence at the department and for anxiety of HCWs. For more details, see Table 3.

**Table 3:** Associations of PSI experience with the hospital management of patient safety culture among HCWs during the second wave of the pandemic; logistic regression models adjusted for age and gender with odds ratio (OR) and 95% confidence interval (95%CI).

Hospital management of patient safety culture	Adjusted Model (age, gender)
	OR (95%CI)
• **Risk of recurrence**	
Expectation of next AE at department in the next 12 months^1^	**2.7(1.42–5.22)**
Expectation of next AE at hospital in the next 12 months^1^	**3.5(1.79–7.02)**
• **Open disclosure experience**	
***Institutional response***	
Reporting and root-cause analysis^2^	2.0(0.92**–**4.10)
Informing and apologising to patient^2^	1.6(0.73**–**3.33)
Conflicts among the colleagues^2^	**2.8(1.17–6.60)**
Losing reputation^2^	2.1(0.64**–**6.84)
Offering institutional support^2^	1.4(0.42**–**4.80)
Buddy support (by colleagues)^2^	1.0(0.53**–**2.02)
***Patient response***	
Accepting explanation and apology^2^	**2.3(1.06–4.81)**
Rejecting the apology and responding aggressively^2^	2.6(0.84**–**7.89)
Patient filed a formal complaint^2^	1.4(0.30**–**6.59)
Patient-HCW relation worsens^2^	2.3(0.88**–**5.98)
• **Second victim experience**	
Sadness^3^	**3.5(1.54–7.86)**
Irritability^3^	**4.3(2.00–9.03)**
Anxiety^3^	**4.1(1.78–9.58)**
Depression^4^	**2.2(1.14–4.06)**

^1^ very likely/somewhat likely; ^2^ always/almost always; ^3^ one or more days; ^4^ mild-severe depression; bold values mean statistically significant, p˂0.05

## Discussion

We found out that one-third of Slovak hospital HCWs experienced at least one PSI and generally reported a poor level of patient safety culture. The majority of them expected the recurrence of AEs at their department and hospital, and few experienced openness in disclosure after the occurrence of AEs. HCWs with PSI experience were more likely to expect subsequent AEs, as well as conflicts among colleagues and patients’ acceptance of an explanation after open disclosure. They were also more likely to have psychological experiences of second victimhood. Finally, most HCWs expressed a high demand to be trained on how to improve their skills to better handle a patient and the aftermath of AEs.

We found that HCWs experiencing a PSI expected another incident more frequently, suggesting underlying organisational issues, as reported by another Slovak study, that frequency of events reported was negatively associated with hospital management support and supervisors’ activity [11]. Previous studies have shown a series of factors that increase the likelihood of recurrence of PSI [25–34]. The main underlying factors for PSI regard human and system (organisational) factors. Important human factors are fatigue, stress, depression, workload and inadequate knowledge or experience [25–30, 35]. Important system factors are lack of supervision, inadequate staffing, number of patients, working hours, missing electronic system, poor teamwork/communication, and distractions [25, 28, 30–34, 36]. Evidence shows that hospital management often fails to take effective actions to prevent the recurrence of some PSI, i.e. as low as 15% [37], though addressing such organisational factors have been shown to be far more effective in prevention than intervening on human factors [38]. All the mentioned factors could have been worsened by the pandemic, during which we collected data. We deduce from our results that hospital management needs to take care of an appropriate patient safety workplace culture and implement preventive actions that address system factors, especially during a pandemic.

We found that experiencing a PSI was associated with conflicts among colleagues, instead of support, after open disclosure. This might be due to ineffective teamwork communication, with mutual blaming and infighting about responsibility for PSI occurrence or even about reporting the PSI. This aligns with the findings of Heard et al. (2012) where about half of HCWs reported those involved in an incident to be blamed and feel unsupported by their colleagues [39]. It also aligns with another study reporting conflicts were also due to the reluctance of some HCWs to disclose, while others wanted to do so [40]. Thus, we propose a “No blame, no shame culture” to be implemented by hospital management [41] as well as team communication skills, and the protocol for PSI reporting and related responsibilities should be transparent.

We found that experiencing a PSI was associated with a patient’s acceptance of an explanation and apology after open disclosure, while other patient responses were not associated. This is in line with another study, which found that openness and provision of adequate information makes patients less likely to file formal complaints towards HCWs or to sue them [42]. Patients are more likely to understand and accept an explanation and apology in PSI occurrence rather than reject it and file complaints or worsens the relations with HCWs. In another study, researchers found that patients just want to be involved in analysing incidents, because they can provide key and reliable information. They need to have a timely and coordinated response to their concerns, which should not stop after providing an explanation [42]. This shows that hospital management should also cultivate an “open culture” at the workplace and support HCWs in speaking up and disclosing to patients, followed by their involvement in further steps of analysis.

We also found that PSI experiences impact the mental well-being of HCWs, fostering psychological consequences of distress, such as irritability, anxiety, sadness, and depression (in order of likelihood). This is in line with findings of Jose Mira et al. (2015) where HCWs also reported other psychological responses, such as guilt, anger, re-living the event, tiredness, insomnia, insecurity, and loss of confidence, which might affect their clinical judgement and lead to their burnout [11, 21]. This all might reflect a psychologically unsafe work environment that requires interventions to enhance resilience, whereas their resilience is currently seen only as a personal issue rather than an organisational issue, too [9, 43]. We learned that the mental health of HCWs is truly affected by the occurrence of AEs and that the resilience of HCWs should be strengthened.

We found a high demand among HCWs for training in topics related to PSI, which confirms the findings of Kalánková et al. (2020) who showed the number of AE significantly depends on the level of education and experience of HCWs [13]. Another study assessing patient safety culture at hospitals found that nurses had a higher level of patient safety culture when they shared information on event reporting, received feedback and had better learning opportunities [12]. This suggests that if offered, HCW training specifically oriented on patient safety culture is likely to be used.

### Strengths and limitations

The main strength of this study is that we were able to approach a representative sample of HCWs from COVID-related departments during the second wave of the pandemic, who admit their experience with PSI. As a result, we could collect information about their PSI experience, and about hospital management of patient safety culture, such as risk of recurrence, open disclosure, and SV experience.

Furthermore, some limitations need to be considered. First, our sample came from only one hospital and was relatively small. By targeting one hospital intensively, we were able to cover a full variety of departments and specialists involved in the pandemic. Nonetheless, our findings probably still reflect an underestimation of the real effects when considering that those experiencing a higher burden might participate less in research. Second, our study had a cross-sectional design, so no causal relations can be provided between PSI experience and hospital management of patient safety culture. Third, we mostly used self-developed questions, which added to the study’s acceptance but may have contributed to some underreporting.

### Implications

Our findings show the problem of adequately handling PSI and developing more openness to be quite big, in particular during the pandemic. This requires measures to improve the patient safety culture to become more open for speaking up, unprejudiced and non-punitive [44].

Second, our findings suggest that currently this problem is managed relatively poorly, implying that better hospital management can make a change here. The strategy needs to be changed from “one of knowledge” to “one of problem solving” [6]. We should be very effective in establishing preventive actions and preparing HCWs guidelines, not only about reporting but also about the process after (how to disclose AEs, how to involve patient and family in the whole process, how to cope with psychological afterwards, how to feel legally protected). This should include near misses [45].

Third, HCWs were very positive about better training, which implies that a fast route to improve the situation should be through their willingness. There are many interesting training courses/programmes/campaigns, which have been successful and we might use as inspiration [46–48]. Regarding this, programmes like “Freedom to Speak Up Guardian”, running in England, could be an example. In that programme the selected HCWs are trained for the position as guardians (“mediators”) who support all other workers to speak up about their concerns and assure that issues are resolved, and all involved in the process receive the solution and feedback [46]. Another example is regulations in the USA, in which the apologies of HCWs are legally protected; this motivates them to apologise more and act transparently with patients [49]. Training is needed for hospital management, as well, especially with a focus on communication skills with case simulations [50, 51].

## Conclusions

HCWs experienced quite a lot of PSI, and those who experienced PSI reported poor hospital management of the patient safety culture and mostly expected a recurrence of AEs; they also reported conflicts related to PSI among colleagues and a negative psychological impact. This shows a need for better management of PSI, both during the COVID-19 pandemic but also thereafter.

### Electronic supplementary material

Below is the link to the electronic supplementary material.

Supplementary Material 1

## Data Availability

The data that support the findings of this study are available from the corresponding author, upon reasonable request. The used questionnaire is attached as Appendix A.
