# Supplementary material for: Opportunities to strengthen resilience of health care workers regarding patient safety
Source: BMC Health Serv Res. 2023 Oct 19;23:1127. doi: 10.1186/s12913-023-10054-0 (PMC10588085; doi:10.1186/s12913-023-10054-0)
Supplement: Supplementary file 1 — Supplementary Material 1 [file 12913_2023_10054_MOESM1_ESM.docx]

# Appendix A – questionnaire used to collect data among Slovak healthcare workers during the second wave of COVID-19.

**Patient Safety Incident Experience (PSI Experience)**

Q.1 Have you seen or heard about “near misses” at your department/hospital in the last year?

(yes/no)

Q.2 Have you seen or heard about “adverse events” at your department/hospital in the last year?

(yes/no)

**Patient Safety Culture**

***Risk of Recurrence***

Q.1 How probable do you think it is that there will be another AE with serious consequences at your department in the next 12 months? (very likely/somewhat likely/unlikely)

Q.2 How probable do you think it is that there will be another AE with serious consequences at your hospital in the next 12 months? (very likely/somewhat likely/unlikely)

***Open Disclosure Experience***

*Institutional Response*

Q1. Please, indicate what followed after the AE occurrence from your own experience or what you heard: (never/sometimes/almost always/always/does not concern me)

- - Reporting/root-cause analysis
  - Informing/apologising to patient (or related family)
  - Offering institutional support (psychological counselling, time off work, training)
  - Conflicts among colleagues (disagreement/criticism)
  - Loss of professional reputation
  - Buddy support

*Patient Response*

Q2. Please, indicate what the consequences were of informing a patient about an AE that affected him/her? (never/sometimes/almost always/always/does not concern me)

- - Patient accepted explanation and apology
  - Relationship with patient worsened as result of the conversation
  - Patient filed a formal complaint
  - Patient rejected the apology and responded aggressively

***Second Victim Experience***

*Sadness/Irritability/Anxiety*

Q1. In the last 6 months: How often did you have:

(every day/more than once a week/every week/every month/rarely/never)

- Sadness
- Irritability
- Anxiety

*Suicidal ideation*

Q.1 How many times in the last week do you have thoughts that it would be better off dead or of hurting yourself in some way:

(several days/more than a half the days/nearly every day vs. not at all)

*Depression (PHQ-19 questionnaire)*

Q.1 Over the *last 2 weeks*, how often have you been bothered by any of the following problems?

(not at all/several days/more than half of the days/nearly every day)

- Little interest or pleasure in doing things
- Feeling down, depressed, or hopeless
- Trouble falling or staying asleep, or sleeping too much
- Feeling tired or having little energy
- Poor appetite or overeating
- Feeling bad about yourself-or that you are a failure or have let yourself or your family down
- Trouble concentrating on things, such as reading the newspaper or watching television
- Moving or speaking so slowly that other people could have noticed? Or the opposite-being so fidgety or restless that you have been moving around a lot more than usual
- Thoughts that you would be better off dead or of hurting in some way

***Promotion of training***

Would you be interested in receiving the specific training? (Yes vs. No)

- 1. How to communicate a PSI.
  2. How to handle an uncooperative or aggressive patient.
  3. How to inform patients or family about AEs.
  4. How HCWs could better cope with the aftermath of AEs.
